# Strain Imaging for the Early Detection of Cardiac Remodeling and Dysfunction in Primary Aldosteronism

**DOI:** 10.3390/diagnostics12020543

**Published:** 2022-02-20

**Authors:** Yilin Chen, Tingyan Xu, Jianzhong Xu, Limin Zhu, Dian Wang, Yan Li, Jiguang Wang

**Affiliations:** 1 Department of Cardiovascular Medicine, Ruijin Hospital, Shanghai Jiaotong University School of Medicine, Shanghai 200025, China; linlin7_cyl@hotmail.com (Y.C.); xjz11691@rjh.com.cn (J.X.); zhulimin@rjh.com.cn (L.Z.); dianwangseu@126.com (D.W.); liyanshcn@yahoo.com (Y.L.); jiguangwang@aim.com (J.W.); 2 State Key Laboratory of Medical Genomics, Shanghai Institute of Hypertension, Ruijin Hospital, Shanghai Jiaotong University School of Medicine, Shanghai 200025, China; 3 Shanghai Key Laboratory of Hypertension, Shanghai Institute of Hypertension, Ruijin Hospital, Shanghai Jiaotong University School of Medicine, Shanghai 200025, China; 4 Center for Cardiovascular Evaluations, Shanghai Institute of Hypertension, Ruijin Hospital, Shanghai Jiaotong University School of Medicine, Shanghai 200025, China; 5 National Research Center for Translational Medicine, Ruijin Hospital, Shanghai Jiaotong University School of Medicine, Shanghai 200025, China

**Keywords:** speckle tracking echocardiography, primary aldosteronism, cardiac dysfunction

## Abstract

Speckle tracking echocardiography is a novel technique to quantify cardiac function and deformation. It has been applied in a series of cardiovascular diseases for the evaluation of early cardiac impairment. We recently used this technique to investigate cardiac structure and function in patients with primary aldosteronism. Cardiac damage usually occurs earlier in patients with primary aldosteronism than those with primary hypertension, probably because aldosterone hypersecretion is more commonly observed in the former than the latter patients. In this article, we will review the imaging studies, especially with speckle tracking echocardiography, for the detection of early cardiac dysfunction in primary aldosteronism as a disease model.

## 1. Introduction

Speckle tracking echocardiography is a novel technique used to quantify cardiac function and deformation [1]. Unlike color or tissue Doppler echocardiography, that may be influenced by the directions of ultrasound beams, this angle-independent technique allows non-invasive structural and functional assessment of both left and right ventricles and atria [2,3]. One of the advantages of this technique, in comparison with the standard echocardiography and tissue Doppler echocardiography, is for the detection of early cardiac dysfunction, especially with the longitudinal strain analysis [2,4]. Indeed, with the conventional standard echocardiography, it is possible to measure left ventricular mass and ejection fraction and define left ventricular hypertrophy and systolic dysfunction. With the use of pulse wave and tissue Doppler echocardiography, it is possible to measure early to late diastolic transmitral flow velocity (E/A) and E to early diastolic mitral annular tissue velocity (E/e’), respectively, as measures of left ventricular diastolic dysfunction [5,6]. These measurements may show late or even terminal lesions of the heart. At these stages, the lesions are often no longer modifiable or are difficult to treat. Speckle tracking echocardiography measures early cardiac impairment in systolic and diastolic function and paves the way for prevention. Strain parameters, especially the global longitudinal strain, are recommended in the evaluation of cardiac function in the guidelines of adult hypertension [7,8]. It may distinguish different etiologies of left ventricular hypertrophy [7] and provide incremental prognostic value upon ejection fraction in the prediction of major adverse cardiac events, especially in patients with near-normal ejection fraction [8].

We recently used this novel technique to investigate early cardiac functional impairment in patients with primary aldosteronism. Probably because of hyperaldosteronism, cardiac dysfunction develops earlier and is often more severe in patients with primary aldosteronism than those with primary hypertension at very similar levels of blood pressure [9,10,11,12]. With the increasing knowledge and improving technology, primary aldosteronism is increasingly diagnosed in patients with hypertension, especially in those with hypokalemia and resistant hypertension. Primary aldosteronism is a common cause of secondary hypertension ranging from 5% in primary care setting [13,14], to 15% in resistant hypertension [15,16] and to 20% in hypertensive patients with other comorbidities, such as diabetes mellitus [17]. In this article we will review the imaging studies, especially with speckle tracking echocardiography, on cardiac remodeling and dysfunction in primary aldosteronism.

## 2. Diagnosis and Screening of Primary Aldosteronism

Hypertension, increased adrenal aldosterone secretion and suppressed renin are the three hallmarks of primary aldosteronism. According to current recommendations, primary aldosteronism is usually screened in patients with stage 2 and 3 hypertension and treatment-resistant hypertension, hypertension plus spontaneous or diuretic-induced hypokalemia, hypertension plus adrenal incidentaloma, hypertension plus a family history of early-onset hypertension or cerebrovascular disease (<40 years), or hypertension plus obstructive sleep apnea and in hypertensive first-degree relatives of patients with primary aldosteronism [18,19,20].

After the withdrawal of mineralocorticoid receptor antagonists, angiotensin-converting enzyme inhibitors, angiotensin receptor blockers, β blockers and diuretics for at least 4 weeks, plasma aldosterone concentration and plasma renin activity are measured after overnight fasting and 30 min of rest in the supine and standing positions. An aldosterone-to-renin ratio of at least 240 pg/mL per ng/mL/h and a plasma aldosterone concentration of at least 200 pg/mL are considered positive for primary aldosteronism. A physiological saline infusion test is then performed to confirm the diagnosis of primary aldosteronism, with a cut-off value of at least 100 pg/mL of plasma aldosterone concentration. Other optional confirming tests include the captopril challenge test and the fludrocortisone suppression test [21]. An adrenal venous sampling procedure and adrenal computed tomography imaging are performed for the subtyping of primary aldosteronism including aldosterone-producing adenoma and idiopathic hyperaldosteronism [18,19,22].

## 3. Left Cardiac Evaluation

### 3.1. Left Ventricular Remodeling

Cardiac remodeling such as left ventricular hypertrophy imposes a definite and independent risk of increased cardiovascular mortality and morbidity in hypertensive patients [23]. It can be evaluated conveniently with conventional echocardiography. In 1996, Rossi GP et al. found a significant increase in left ventricular mass index and higher prevalence of left ventricular hypertrophy in patients with primary aldosteronism compared with well-matched primary hypertensive patients [9]. Similar results were later observed in the comparison between patients with the aldosterone-producing adenoma type of primary aldosteronism and those with primary hypertension [24]. The left ventricular wall thickness was positively and independently correlated with the aldosterone level [9] and 24 h urinary sodium excretion [25]. A study of Muiesan ML et al. showed that patients with primary aldosteronism had a significantly increased prevalence of inappropriate left ventricular mass even in the absence of traditionally defined left ventricular hypertrophy [8], possibly indicating an accelerated phase of transition from compensatory left ventricular hypertrophy toward heart failure [26]. In addition, left ventricular hypertrophy in patients with primary aldosteronism was more prominent than in patients with other secondary forms of hypertension, probably due to the abnormal hypersecretion of aldosterone [11,27].

### 3.2. Systolic Dysfunction

In early studies of primary aldosteronism, though left ventricular hypertrophy has been investigated, systolic functional evaluation was limited to left ventricular ejection fraction or fractional shortening measured by standard echocardiography. In most of the recent studies, left ventricular ejection fraction was similar in patients with primary aldosteronism and those with primary hypertension or normotension [28]. Only in the study of Muiesan ML et al., mid-wall fractional shortening was found significantly lower than in primary hypertensive patients [10]. The cyclic variation of integrated backscatter and strain analysis by tissue Doppler were then adopted in several clinical studies with significant decreases in patients with primary aldosteronism or its subtype aldosterone-producing adenoma [29,30,31,32]. Although both techniques provide additional information on cardiac systolic function, the entire segments cannot be fully evaluated with these two methods because of the angle- or view-dependence disadvantage [5,33].

**Figure 1:**
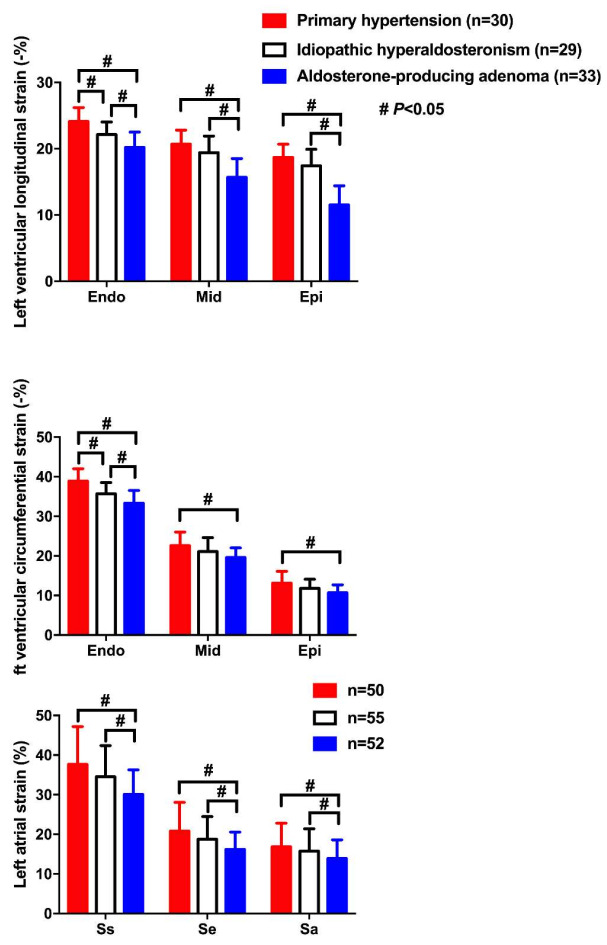
Left ventricular longitudinal (**upper**) and circumferential (**middle**) layer strain analyses and left atrial strain (**lower**) analyses in patients with primary hypertension (red bars), idiopathic hyperaldosteronism (white bars) and aldosterone producing adenoma (blue bars). Symbols represent mean and vertical lines denote standard deviation. Ss—left atrial strain during reservoir period; Se—left atrial strain during conduit period; Sa—left atrial strain during contractile period. The number of patients among the three groups are given. Reproduced with permission from Wang D, et al. [39,50].

Speckle tracking echocardiography overcomes this disadvantage and has been recently used in the evaluation of systolic function in primary aldosteronism. Chen ZW et al. first reported that patients with primary aldosteronism had a significantly lower left ventricular global longitudinal strain than patients with primary hypertension (−17.8 ± 2.4 vs. −20.3 ± 2.3%, *p* < 0.001), although the two groups had similar left ventricular ejection fraction [34]. Layer-specific strain analysis, derived from speckle tracking echocardiography, quantifies multi-layer myocardial function including endocardium, midmyocardium and epicardium [35] and provides more detailed information on myocardial motion [36]. At the early stages of hypertension, endocardium seems to be the most affected by pressure overload [37], while epicardium is involved in more advanced stages and is described as a predictor of cardiovascular events [38]. Using this technique, we studied the two major subtypes of primary aldosteronism, aldosterone-producing adenoma and idiopathic hyperaldosteronism, and found that the left ventricular longitudinal and circumferential strain in endocardium, midmyocardium and epicardium was lowest in aldosterone-producing adenoma, intermediate in idiopathic hyperaldosteronism and highest in primary hypertension (*p* < 0.001, Figure 1) [39]. The difference between these three groups was more prominent for the epicardium than for the endocardium and the midmyocardium. The decrease in both longitudinal and circumferential layer-specific strains was correlated with plasma aldosterone concentration (r = −0.69 to −0.38, *p* < 0.001). The correlation also tended to be stronger for the epicardium than for the endocardium and the midmyocardium.

### 3.3. Diastolic Dysfunction

Left ventricular diastolic function usually includes the assessment of the left atrial size and Doppler measurements of E/A and e’/a’ [6]. The significant difference in the E/A ratio was observed in early studies of primary aldosteronism, suggesting the adverse effect of aldosterone on diastolic function [24,31,40]. However, evidence has shown that the E/A ratio is less reliable for evaluating diastolic function as it might be influenced by preload and age. E’ assessed by tissue Doppler is less load dependent and is used to indicate left ventricular filling pressure when combined with E velocity according to current guidelines [6]. In a previous study of Yang Y et al., a decrease in e’, an increase in E/e’ and left atrial enlargement were observed in patients with primary aldosteronism [12]. In addition, Chang YY et al. further demonstrated the significant and independent correlation between 24 h urinary aldosterone excretion and diastolic function as assessed by E/e’ [41].

Speckle tracking echocardiography has been recently found to have potential usefulness and clinical relevance in the detection of left ventricular diastolic dysfunction by adding left atrial strain to left atrial size [42,43]. It seems to besuperior to conventional echocardiography in the assessment of left atrial function, especially in the evaluation throughout the cardiac cycle including left atrial reservoir, conduit and contractile periods [44,45,46]. Indeterminate diastolic function would occur with conventional echocardiography. Left atrial strains, especially the reservoir strain, could improve the assessment of diastolic function [47] and significantly correlate with left ventricular filling pressure [48]. Thus, a recent expert consensus document included these left atrial strain measurements in the evaluation of diastolic dysfunction in heart failure with preserved ejection fraction [49]. Using this technique, we found that patients with aldosterone-producing adenoma had a significantly lower strain and strain rate in the left atrium during atrial reservoir, conduit and contractile phases than patients with idiopathic hyperaldosteronism and primary hypertension (Figure 1) [50]. These left atrial strain parameters were significantly correlated with both plasma and urinary aldosterone level with a correlation coefficient of −0.17 to −0.38 (*p* ≤ 0.03).

## 4. Right Cardiac Evaluation

In the past several decades, right cardiac evaluation has become increasingly important in many cardiovascular diseases [51] and even in the general population [52]. Indeed, right ventricular dysfunction could also be associated with hypertension as both the right and left ventricles share the interventricular septum and it tended to be a remarkable predictor of heart failure associated with hypertension [53]. Right ventricular hypertrophy was found commonly in hypertensive patients [54] with a significant decrease in right ventricular diastolic function compared with normotensive subjects [55]. Pulmonary vascular resistance, measured by mean pulmonary arterial pressure, increases in uncomplicated hypertension, suggesting an early pulmonary vascular remodeling [56].

Hyperaldosteronism might further aggravate right ventricular dysfunction, which has been shown in studies with conventional echocardiography, when measured as right ventricular myocardial performance index (RIMP) and tricuspid annular plane systolic excursion (TAPSE) [57,58]. Right ventricular–pulmonary arterial uncoupling can be assessed as the ratio of TAPSE to pulmonary arterial systolic pressure. This ratio has been reported to be a useful clinical index of right ventricular dysfunction and may predict clinical outcomes in patients with heart failure [59,60]. This ratio also markedly decreased in hypertensive patients without heart failure [61]. Though these conventional measurements provide information on the evaluation of right cardiac function, it should be noted that TAPSE represents the contraction of the basal segment of the right ventricular free wall instead of the entire right ventricle [62]. RMIP, although considered as one of the most accurate indexes of ventricular systolic and diastolic function, yet is still load dependent and, when right atrial pressure is elevated, unreliable [63].

Speckle tracking echocardiography also offers advantages in the evaluation of right cardiac function. Indeed, right ventricular strain derived from speckle tracking echocardiography could be a better approach in the assessment of right cardiac function. Tadic M et al. found significantly lower right ventricular strains including both the four-chamber and free wall longitudinal strain with the application of speckle tracking echocardiography in patients with either isolated night-time hypertension or daytime and night-time hypertension than normotensive control subjects and patients with isolated daytime hypertension [64]. Besides, in their study, the decrease of right ventricular strain was more deteriorated in the non-dipper than dipper hypertensive patients [65] and significantly correlated with right ventricular deformation [64].

**Figure 2:**
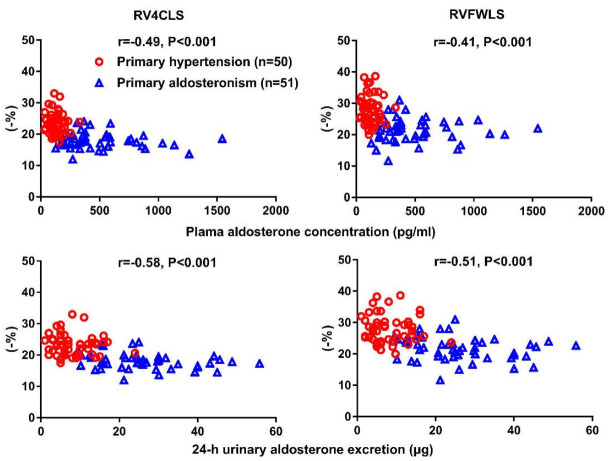
Correlation analyses of right ventricular four-chamber longitudinal strain (RV4CLS, **left**) and right ventricular free wall longitudinal strain (RVFWLS, **right**) with plasma aldosterone concentration (**upper**) and 24 h urinary aldosterone excretion (**lower**) in patients with primary aldosteronism (triangle) and those with primary hypertension (circle). The correlation coefficients and corresponding *p* values are given for all patients. Reproduced with permission from Chen YL, et al. [66].

With the new technique, we recently found that patients with primary aldosteronism had significantly (*p* < 0.001) lower right ventricular four-chamber and free wall longitudinal strain (−18.1 ± 2.5% and −21.7 ± 3.7%, respectively) than patients with primary hypertension (−23.3 ± 3.4% and −27.9 ± 4.5%, respectively), in addition to significant right cardiac enlargement and pulmonary arterial pressure elevation and in spite of similar and normal right ventricular ejection fraction [66]. In both groups combined, the right ventricular strains were significantly correlated with plasma and urinary aldosterone level with a correlation coefficient of −0.41 to −0.58 (*p* < 0.001, Figure 2). Taking the results of these cardiac studies together, it is clear that the speckle tracking echocardiographic technique can show earlier functional impairment in the left as well as the right heart.

## 5. Cardiac Myocardial Work Evaluation

Strain analysis is useful in the quantitative assessment of cardiac functional performance. However, strain parameters are still load dependent [67] as evidence that the decrease of strain might also be affected by the elevation of left ventricular afterload [68]. An even newer technique, the left ventricular pressure–strain loop, which is based on speckle tracking echocardiography and cardiac afterload [69], may provide more information on cardiac physiological changes under various loading conditions than global longitudinal strain in cardiac evaluation [70]. This advanced technique included four major myocardial work indices: global myocardial work index (GWI), global constructive work (GCW),global wasted work (GWW) and global work efficiency (GWE). In brief, GWW and GWE might act as indices of energy loss to indicate dyssynchrony and remodeling [71,72], while GWI and GCW are more related to the total energy and oxygen consumption at various after loads [73]. These indices have shown advantages in the early diagnosis of coronary artery disease [74] and the prediction of cardiac resynchronization therapy response over global longitudinal strain [75].

This novel method has recently been adopted in several studies on hypertension. Chan J et al. first investigated myocardial work analysis in hypertensive patients and found that GWIand GCW were comparable in patients with stage 1 hypertension and normotensive control subjects, but significantly increased in patients with stage 2 and 3 hypertension [76], though GWW and GWE remained similar among all groups. Jaglan A et al., on the other hand, found a significant increase in GWW in addition to the similar significant differences in GWI and GCW in patients with stage 1 and 2 hypertension and normotensive subjects [77]. Nonetheless, it should be noted that the global longitudinal strain in these studies were all comparable between groups, suggesting that these patients were in the early stage of cardiac remodeling and dysfunction. Indeed, Sahiti F et al. found that hypertension was associated with a mild increase in GCW, but a profound increase in GWW, resulting in a higher GWI and a lower GWE [78].

**Figure 3:**
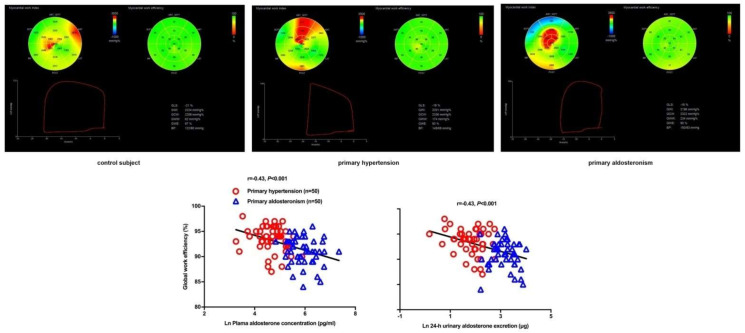
Myocardial work analysis in a control subject (**upper left**), primary hypertensive patient (**upper middle**) and primary aldosteronism patient (**upper right**) and the correlation analysis between global work efficiency with plasma aldosterone concentration (**lower left**) and urinary aldosterone excretion (**lower right**). The correlation coefficients and corresponding *p* values are given for all subjects. Reproduced from Chen YL, et al. [79].

**Figure 4:**
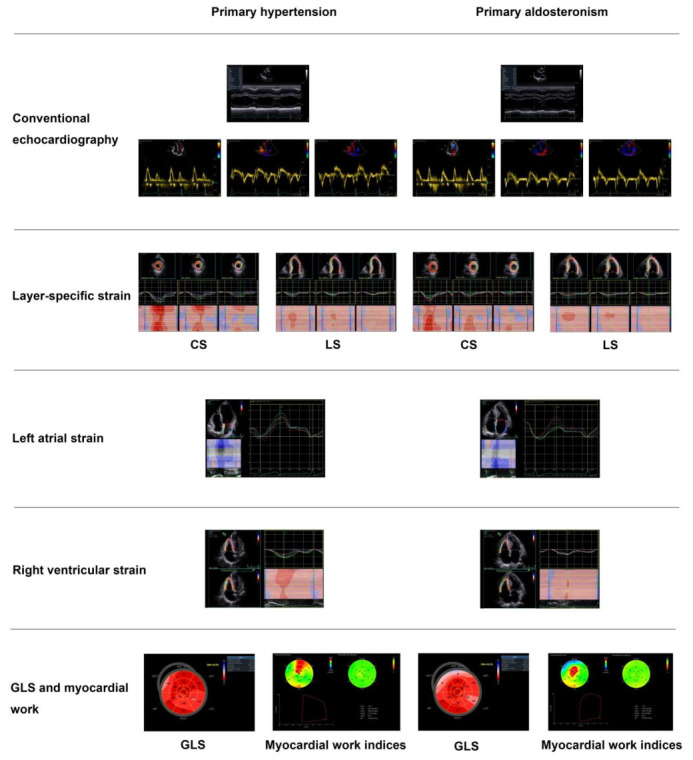
Main echocardiographic findings in primary hypertension and primary aldosteronism. Two representative cases of different echocardiographic parameters in a patient with primary hypertension (**left panels**) and in another with primary aldosteronism (**right panels**) with similar blood pressure. CS—circumferential strain; GLS—global longitudinal strain; LS—longitudinal strain. Values in the figure are expressed as below. Blood pressure: 149/69 in the primary hypertensive patient vs. 150/83 mmHg in the primary aldosteronism patient; left ventricular mass index: 97 vs. 145 g/m^2^, respectively; E/A: 1.4 vs. 0.88, respectively; E/e’: 10.4 vs. 18.9, respectively; CSendo (basal short-axis view): −42.8 vs. −39.2%, respectively; CSmid: −26.3 vs. −21.5%, respectively; CSepi: −13.6 vs. −10.4%, respectively; LSendo (four-chamber view): −21.4 vs. −19.9%, respectively; LSmid: −19.0 vs. −16.4%, respectively; LSepi: −16.8 vs. −13.3%, respectively; LASs (left atrial strain, four-chamber view): 27.8 vs. 20.4%, respectively; LASe: 17.6 vs. 10.7%, respectively; LASa: 10.3 vs. 9.8%, respectively; right ventricular four-chamber longitudinal strain: −24.6 vs. −17.3%, respectively; GLS: −18.5 vs. −16.2%, respectively; global myocardial work index: 2201 vs. 2196 mmHg%, respectively; global constructive work: 2336 mmHg% vs. 2322 mmHg%, respectively; global wasted work: 174 vs. 234 mmHg%, respectively; global work efficiency: 93 vs. 90%, respectively.

With this novel technique, we found that patients with primary aldosteronism had a significantly decreased GWE, but similar GWI, in comparison with primary hypertensive patients and normotensive subjects (Figure 3) [79]. We speculated that similar and normalized total myocardial work could possibly be a compensation at the expense of work efficiency. Furthermore, we found that the global work efficiency was also significantly correlated with the plasma urinary aldosterone level (r = −0.43 for both, *p* < 0.001, Figure 3), independent of left ventricular mass index and blood pressure. A summary figure of main echocardiographic findings is presented in Figure 4 between patients with primary hypertension and those with primary aldosteronism.

## 6. Myocardial Fibrosis

In addition to left ventricular hypertrophy, hyperaldosteronism has also been shown to be associated with myocardial fibrosis [80], which might further worsen cardiac diastolic and systolic function, impact cardiac remodeling and increase ventricular stiffness [81]. Cardiac magnetic resonance (MR) imaging has been an established reference imaging method for the assessment of cardiac anatomy and function [82]. According to the cardiomyopathic process, myocardial fibrosis can be classified as replacement, diffuse interstitial and infiltrative interstitial fibrosis [81,83]. The first two types of fibrosis are usually presented in hypertensive patients and could be detected by cardiac MR imaging. Myocardial replacement fibrosis is characterized by increased extracellular volume distribution that may cause late gadolinium enhancement (LGE) [84]. It is considered as the later stage of disease when cellular damage and cardiomyocyte necrosis have appeared [85]. In an early cardiac MR imaging study, Rudolph A et al., found that the presence of non-infarct LGE patterns was recognized in about 50% of the patients with hypertension-induced left ventricular hypertrophy and was significantly correlated with left ventricular mass index [86]. Diffuse interstitial fibrosis, on the other hand, is another type of myocardial fibrosis seen in hypertensive patients with a diffuse distribution within the interstitium as well as the perivasculature [87], which could not be detected with LGE. Thus, T1 mapping with the evaluation of extracellular volume fraction in cardiac MR imaging was developed as a quantitative technique to identify this type of fibrosis by computing the pre- and post-contrast T1 value of the myocardium and blood pool together with hematocrit [88]. Several studies have demonstrated high values of extracellular volume fraction and native T1 in hypertensive patients, both of which were associated with left ventricular mass and left ventricular hypertrophy [89,90].

Freel EM et al. first investigated cardiac fibrosis in patients with primary aldosteronism using cardiac MR imaging [91]. They found a 4.3 times higher prevalence of non-infarct related replacement fibrosis in patients with primary aldosteronism than those with primary hypertension. Su MY et al., on the other hand, found a significant increase in diffuse fibrosis compared with control subjects, although replacement fibrosis was not measured in their study [92]. In a recent study, extracellular mass index was significantly increased only in primary aldosteronism, while extracellular volume fraction increased in both primary and secondary aldosteronism, possibly because left ventricular structure was associated with primary pathological aldosterone secretion [93]. On the contrary, in another study, Grytass MA et al., did not find any difference in diffuse interstitial fibrosis between the patients with primary aldosteronism and the control subjects [94]. The contradictory results between the two studies might be explained by the difference in the blood pressure level and sample size and by the difference in antihypertensive therapy as mineralocorticoid receptor antagonists or adrenalectomy might affect the fibrosis extent in primary aldosteronism.

Speckle tracking echocardiography might also be useful in the evaluation of myocardial fibrosis. Several previous studies showed good agreement between this echocardiographic technique and cardiac MR imaging. Strain parameters derived from speckle tracking echocardiography have a significant predictive value in the identification of delayed LGE segments and intramyocardial hemorrhage in acute ST-segment elevation myocardial infarction [95,96]. Global longitudinal strain has been shown to be significantly correlated with the degree of replacement fibrosis in patients with dilated cardiomyopathy [97]. Regional longitudinal strain, on the other hand, might be the best indicator to detect segmental fibrosis in patients with hypertrophic cardiomyopathy [98]. Besides, a significant and inverse correlation between left atrial reservoir strain and myocardial fibrosis was also demonstrated by cardiac MR in patients with atrial fibrillation [99]. With the novel left ventricularpressure–strain technique, Galli E et al., found in patients with hypertrophic cardiomyopathy that GCW, instead of global longitudinal strain, had significant predictive value for left ventricular fibrosis [100]. We recently applied this echocardiographic technique to investigate myocardial fibrosis in primary aldosteronism, with cardiac MR imaging as standard. We found that GWE was associated with focal replacement fibrosis as assessed by cardiac MR imaging, while another fibrosis type, diffuse interstitial fibrosis, was associated with abnormalities in aldosterone and renin [101].

## 7. Treatment Effect on Cardiac Structure and Function

Current clinical guidelines recommend adrenalectomy for unilateral primary aldosteronism and medical treatment for bilateral idiopathic hyperaldosteronism orthose who are unwilling to undergo surgery [19,22,102]. Several previous studies have demonstrated that these treatments, especially adrenalectomy, may reverse the cardiac structural alterations such as left ventricular hypertrophy [30,103]. In a prospective study, Lin YH. et al., found a significant regression of left ventricular mass index and a decrease in the cyclic variation of integrated backscatter in patients with adrenalectomy at 1 year of follow-up [29,30]. In a retrospective study of drug treatment, Ori Y. et al., found that left ventricular mass index significantly decreased at 1 year of follow-up and normalized at 3 years [104]. Besides, the regression of left ventricular hypertrophy and the reduction in right ventricular volume measured by cardiac MR were also prospectively observed at 3 and 6 months of follow-up in a drug treatment study by Gaddam K et al. [105]. In a comparison study, Catena C. et al., prospectively found that the response of left ventricular mass occurred earlier in adrenalectomized patients than in those treated with spironolactone but later became comparable in the two groups of patients during an average follow-up of 6.4 years [40]. On the contrary, other studies have shown a significant regression in left ventricular mass in the surgery group, while the medical treatment group showed no change or slightly decreased in cardiac structure [106,107,108]. However, these results should be interpreted with caution, because the clinical characteristics and echocardiographic parameters at baseline and the follow-up time were different.

The surgical and medical treatments not only regressed left ventricular structure, but also improved diastolic function when evaluated by E/e’. Indra T et al. found that E/e’ was significantly decreased in both surgically and medically treated patients during a median follow up of 64 months [109]. Chang YY et al. demonstrated that patients with adrenalectomy had an improvement in diastolic function as reflected by a significant decrease in E/e’ [110]. They further demonstrated that only patients with KCNJ5 mutation, but not those with other mutations, had a significant improvement in diastolic function and regression of left ventricular mass after surgery [111]. A summary table of main imaging studies with conventional echocardiography and cardiac MRon primary aldosteronism is presented in Table 1.

**Figure 5:**
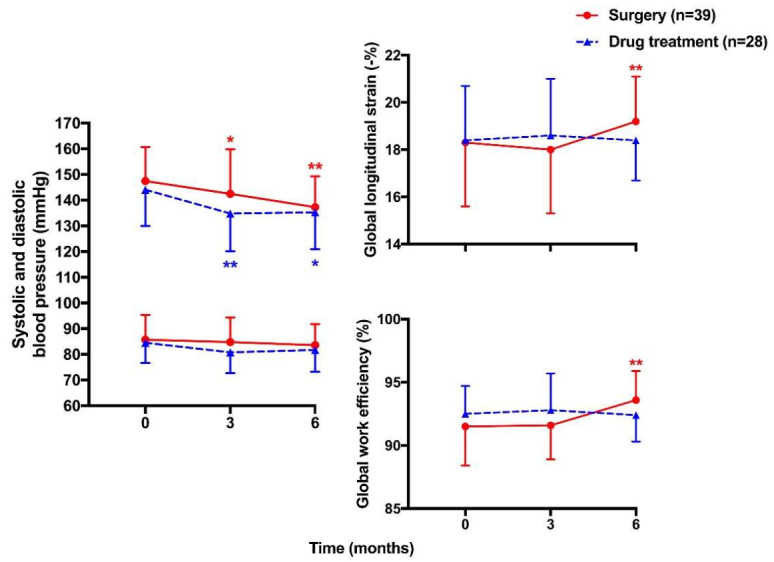
Mean (± SD) values of systolic (**upper left lines**) and diastolic blood pressure (**lower left lines**) and global longitudinal strain (**upper right**) and global work efficiency (**lower right**) of the left ventricle at baseline and during follow-up in patients treated with surgery (circles with solid lines) and drugs (triangles with dashed lines). * *p* < 0.05, ** *p* < 0.01 vs. baseline. Reproduced from Chen YL, et al. [116].

Previous studies rarely investigated treatment effects on alterations in systolic function, possibly because of the limitations of evaluation with conventional echocardiography. Speckle tracking echocardiography has recently been applied in the evaluation of treatment effects of cardiovascular diseases such as coronary artery disease and heart failure [112,113]. Nonetheless, there is limited data on the effect of antihypertensive medications on left ventricular strain, probably because of the difficulties in differentiating the effect of antihypertensive drugs from that of blood pressure reduction [114]. The left ventricular pressure–strain loop analysis might overcome this dilemma and be useful in the evaluation of treatment effects in primary aldosteronism, as this technique takes the left ventricular afterload into consideration. In a recent study, Ikonomidis I et al., found that cardiac function defined as an increase in GCW and a decrease in GWW was significantly improved in diabetic patients treated with combination therapy of two new antidiabetic agents [115]. We used the speckle tracking echocardiographic technique, including global longitudinal strain and myocardial work indices, to investigate treatment effects of adrenalectomy and mineralocorticoid receptor antagonists in patients with primary aldosteronism [116]. In a 6-month follow-up study, we observed that surgery, but not drug treatment, significantly improved global longitudinal strain and GWE; though both treatments effectively lowered blood pressure and normalized serum potassium. The changes in GWE were dependent on the changes in the aldosterone level in patients treated with spironolactone but not those treated with adrenalectomy, indicating that adrenalectomy might have treatment effects over and beyond reductions in the aldosterone secretion and mineralocorticoid receptor antagonism (Figure 5). A summary table of imaging studies with speckle tracking echocardiographyon primary aldosteronism is presented in Table 2.

**Table 1:** Summary of main imaging studies with conventional echocardiography and cardiac magnetic resonance on primary aldosteronism.

First Author	Year	Study Design	Participants	No. of Participants	Age (y)	Sex (Male)	Outcome Measures	Results	Summary of Findings
Rossi GP [9]	1996	Cross-sectional	PA vs. PH	34 vs. 34	51 ± 13 vs. 49 ± 12	18 vs. 18	LVMI	112 ± 5 vs. 98 ± 4 g/m^2^	Significantly greater LVMI and higher prevalence of LVH in PA than PH
Matsumura K [27]	2006	Cross-sectional	PA vs. Renovascular hypertension	25 vs. 29	47 ± 2 vs. 45 ± 4	13 vs. 10	LVMI	154 ± 7 vs. 135 ± 9 g/m^2^	Higher prevalence of LVH in PA than renovascular hypertension
Muiesan ML [10]	2008	Cross-sectional	PA vs. PH	125 vs. 125	50 ± 11 vs. 51 ± 11	71 vs. 71	LVMI	50 ± 17 vs. 40 ± 11 g/m^2.7^	Significantly higher prevalence of inappropriate LVMI in the absence of traditionally defined LVH in PA than PH
Yang Y [12]	2017	Cross-sectional	PA vs. PH	100 vs. 100	50 ± 12 vs. 50 ± 12	58 vs. 58	LAVI&E/e’	LAVI: 23 ± 6 vs. 21 ± 6 mL/m^2^ E/e’: 13.5 ± 4.3 vs. 11.9 ± 3.3	Significantly lower e’ and higher E/e’ in PA than PH, in addition to left atrial enlargement
Catena C [40]	2007	Prospective longitudinal	PA	Surgery 24 vs. Drug treatments 30	53 ± 12 in PA patients	38 inPA patients	LVMI	53 ± 11 vs. 52 ± 11 g/m^2.7^ at baseline 45 ± 12 vs. 49 ± 11 g/m^2.7^ at 1 year 43 ± 11 vs. 44 ± 11 g/m^2.7^ at the end of study	Earlier response of LVM regression in surgery than drug treatment but later comparable in the two groups during an average of 6.4 years follow-up
Lin YH [29]	2011	Prospective longitudinal	PA	Surgery 11	47 ± 8	5	LVMI	153 ± 31 at baseline vs. 116 ± 12 g/m^2^ at 1 year	Significant regression in LVMI at 1 year
Ori Y [104]	2013	Retrospective	PA	Drug treatment 48	61 ± 10	28	LVMI	142 ± 28 at baseline, 121 ± 21 at 1 year and 112 ± 24 g/m^2^ at 3 years	Significant decrease in LVMI at 1 year and normalized at 3 years
Rossi GP [106]	2013	Prospective longitudinal	PA	Surgery 110 vs. Drug treatment 70	51 ± 12 in PA patients	57 in PA patients	LVMI	53 ± 13 vs. 50 ± 11 g/m^2.7^ at baseline 49 ± 10 vs. 47 ± 8 g/m^2.7^ during follow-up	Significant regression in LVMI in surgery but with slight decrease in drug treatment at a median of 36 months follow-up
Indra T [111]	2015	Prospective longitudinal	PA	Surgery 15 vs. Drug treatment 16	49 ± 11 vs. 51 ± 7	9 vs. 11	LVMI&E/e’	LVMI: 50 ± 12 vs. 53 ± 12 g/m^2.7^ at baseline 39 ± 9 vs. 49 ± 11 g/m^2.7^ at the end of study E/e’: 9.6 ± 3.0 vs. 14.4 ± 4.4 at baseline 7.1 ± 1.1 vs. 8.7 ± 1.0 at the end of study	Significant decrease in E/e’ in both surgery and drug treatment groups, with regression of LVMI only in surgery group
Freel EM [91]	2012	Cross-sectional	PA vs. PH	27 vs. 53	54 ± 11 vs. 55 ± 9	21 vs. 42	LGE	70% vs. 13%	4.3 times higher prevalence of non-infarct related replacement fibrosis in PA than PH
Su MY [92]	2012	Cross-sectional	PA vs. Controls	25 vs. 12	50 ± 13 vs. 49 ± 14	6 vs. 7	EV	0.43 ± 0.05 vs. 0.36 ± 0.07	Significantly increased diffuse fibrosis in PA compared with controls

Values are expressed as mean ± SD or percentage of patients. E—the peak early filling velocity of transmitral flow; E’—the average peak early filling velocity of septal and lateral mitral annulus; EV—enhancement value; LGE—late gadolinium enhancement; LAVI—left atrial volume index; LVH—left ventricular hypertrophy; LVMI—left ventricular mass index; PA—primary aldosteronism; PH—primary hypertension.

**Table 2:** Summary of main imaging studies with speckle tracking echocardiography on primary aldosteronism.

First Author	Year	Study Design	Participants	No. of Participants	Age (y)	Sex (Male)	Outcome Measures	Results	Summary of Findings
Chen ZW [34]	2018	Cross-sectional	PA vs. PH	36 vs. 31	49 ± 11 vs. 53 ± 12	15 vs. 16	GLS	−17.8 ± 2.4 vs. −20.3 ± 2.3%	Significantly lower GLS in PA than PH
Wang D [39]	2019	Cross-sectional	APA, IHAand PH	33, 29 and 30	49 ± 10, 52 ± 8 and 51 ± 20	20, 22 and 18	LS&CS	LSendo: −20.2 ± 2.3, −22.1 ± 1.9 and −24.1 ± 2.1% LSmid: −15.7 ± 2.8, −19.4 ± 2.5 and −20.7 ± 21% LSepi: −15.8 ± 2.1, −19.6 ± 2.2 and −21.2 ± 1.9% CSendo: −33.3 ± 3.2, −35.7 ± 2.8 and −38.9 ± 3.1% CSmid: −19.6 ± 2.4, −21.1 ± 3.5 and −22.6 ± 3.4% CSepi: −10.7 ± 2.0, −11.8 ± 2.3 and −13.1 ± 3.0%	Lowest CS and LS in endocardium (endo), midmyocardium (mid) and epicardium (epi) in APA, intermediate in IHA, and highest in PH
Wang D [50]	2019	Cross-sectional	APA, IHA and PH	52, 55 and 50	52 ± 11, 51 ± 10 and 50 ± 17	35, 34 and 33	LAS&LASR	LASs: 30.1 ± 6.2, 34.5 ± 7.9 and 37.7 ± 9.5% LASe: 16.2 ± 4.4, 18.8 ± 5.7 and 20.8 ± 7.3% LASa: 13.9 ± 4.7, 15.8 ± 5.6 and 16.9 ± 6.0% LASRs: 1.8 ± 0.6, 1.9 ± 0.4 and 2.1 ± 0.7/s LASRe: 1.6 ± 0.4, 1.7 ± 0.4 and 1.9 ± 0.6/s LASRa: 1.5 ± 0.5, 1.6 ± 0.6 and 1.8 ± 0.5/s	Significantly lower LAS and LASR during atrial reservoir (s), conduit (e) and contractile (a) phases in APA than IHA and PH
Chen YL [66]	2020	Cross-sectional	PA vs. PH	51 vs. 50	51 ± 11 vs. 53 ± 11	34 vs. 30	RV4CLS & RVFWLS	RV4CLS: −18.1 ± 2.5 vs.−23.3 ± 3.4% RVFWLS: −21.7 ± 3.7 vs. −27.9 ± 4.5%	Significantdecrease in both RV4CLS and RVFWLS in PA than PH
Chen YL [79]	2021	Cross-sectional	PA vs. PH	50 vs. 50	51 ± 10 vs. 55 ± 11	32 vs. 33	Strain (GLS) and myocardial work indices (GWI, GCW, GWW, & GWE)	GLS: −18.0 ± 2.1 vs. −19.2 ± 2.0% GWI: 2336 ± 333 vs. 2366 ± 288 mmHg% GCW: 2494 ± 325 vs. 2524 ± 301 mmHg% GWW: 206 ± 75 vs. 142 ± 56 mmHg% GWE: 91.1 ± 2.7 vs. 93.5 ± 2.5%	Significant decrease in GLS and GWE and increase in GWW in PA than PH, with similar GWI and GCW in the two groups
Chen YL [116]	2021	Prospective longitudinal	PA	Surgery 39 vs. Drug treatment 28	49 ± 10 vs. 49 ± 12	26 vs. 22	Strain (GLS) and myocardial work indices (GWI, GCW, GWW, & GWE)	GLS: −18.3 ± 2.7 vs. −18.4 ± 2.3% at baseline 19.2 ± 1.9 vs. 18.4 ± 1.7% at 6 months GWI: 2372 ± 388 vs. 2335 ± 341 mmHg% at baseline 2280 ± 344 vs. 2208 ± 306 mmHg% at 6 months GCW: 2510 ± 360 vs. 2437 ± 293 mmHg% at baseline 2436 ± 335 vs. 2330 ± 311 mmHg% at 6 months GWW: 201 ± 87 vs. 164 ± 56 mmHg% at baseline 142 ± 58 vs. 164 ± 53 mmHg% at 6 months GWE: 91.5 ± 3.1 vs. 92.5 ± 2.2% at baseline 93.6 ± 2.3 vs. 92.4 ± 2.1% at 6 months	Significant improvement in GLS and GWE in surgery but not drug group at 6-month follow-up

Values are expressed as mean ± SD. APA—aldosterone-producing adenoma; CS—circumferential strain; GCW—global constructive work; GLS—global longitudinal strain; GWE—global work efficiency; GWI—global work index; GWW—global wasted work; IHA—idiopathic hyperaldosteronism; LAS—left atrial strain; LASR—left atrial strain rate; LS—longitudinal strain; PA—primary aldosteronism; PH—primary hypertension; RV4CLS—right ventricular four-chamber longitudinal strain; RVFWLS—right ventricular free wall longitudinal strain.

## 8. Biomarkers in Primary Aldosteronism

Plasma aldosterone concentration and plasma renin activity are the major diagnostic biomarkers in primary aldosteronism. Besides, the 24 h urinary aldosterone excretion could also be useful to the diagnosis [18,19,20]. N-terminal pro B-type natriuretic peptide (NT-proBNP) was found significantly higher in patients with primary aldosteronism than those with primary hypertension and was correlated with plasma aldosterone level [117]. In our cardiac MR study, it was associated with both focal replacement and diffuse interstitial fibrosis [101]. During the follow-up study, a reduced pre-treatment plasma aldosterone level after surgical removal of the adrenal gland and increased post-treatment plasma renin activity in the medically treated patients were shown to be possibly predictive of favorable changes in cardiac structure and clinical outcomes [118,119].

## 9. Conclusions and Perspectives

Strain echocardiography is useful in the early detection of cardiac structural and functional alterations in both the left and the right heart. Primary aldosteronism is a very good disease model for the research of this novel technology for several reasons. First, patients with primary aldosteronism develop cardiac structural and functional changes earlier and more severely than those with primary hypertension at similar levels of blood pressure. Second, there are specific treatments, either surgical or medical, for primary aldosteronism. Treatment effects can be studied for both intermediate and hard clinical outcomes. Third, there is increasing awareness of primary aldosteronism. With the use of an automated chemiluminescence technique for the measurement of plasma renin and aldosterone concentration, massive screening is feasible. There would be a huge number of patients with hypertension and primary aldosteronism diagnosed and treated [120].

Strain echocardiography may be increasingly used in the evaluation of cardiac function at various stages of the disease, such as at the diagnosis and after surgical and medical treatment. Nonetheless, this advanced echocardiographic technique in primary aldosteronism has been recently adopted in primary aldosteronism. There are still a number of unresolved research questions, such as its long-term predictive value for major adverse cardiac events. Future research should address whether strain echocardiography may provide prognostic value and guide the choice of treatment modalities, and hence improve clinical outcomes.
